# Water chemistry in 179 randomly selected Swedish headwater streams related to forest production, clear-felling and climate

**DOI:** 10.1007/s10661-014-4054-5

**Published:** 2014-09-27

**Authors:** Stefan Löfgren, Mats Fröberg, Jun Yu, Jakob Nisell, Bo Ranneby

**Affiliations:** 1 Department of Aquatic Sciences and Assessment, Swedish University of Agricultural Sciences, Box 7050, SE-750 07 Uppsala, Sweden; 2 Department of Mathematics and Mathematical Statistics, Umeå University, SE-901 87 Umeå, Sweden; 3 Geological Survey of Sweden, P.O. Box 670, SE-751 28 Uppsala, Sweden; 4 Centre of Biostochastics, Swedish University of Agricultural Sciences, SE-901 83 Umeå, Sweden

**Keywords:** Water chemistry, Headwater streams, Boreal landscape, Forestry, Representative sampling, Probabilistic classifying

## Abstract

From a policy perspective, it is important to understand forestry effects on surface waters from a landscape perspective. The EU Water Framework Directive demands remedial actions if not achieving good ecological status. In Sweden, 44 % of the surface water bodies have moderate ecological status or worse. Many of these drain catchments with a mosaic of managed forests. It is important for the forestry sector and water authorities to be able to identify where, in the forested landscape, special precautions are necessary. The aim of this study was to quantify the relations between forestry parameters and headwater stream concentrations of nutrients, organic matter and acid-base chemistry. The results are put into the context of regional climate, sulphur and nitrogen deposition, as well as marine influences. Water chemistry was measured in 179 randomly selected headwater streams from two regions in southwest and central Sweden, corresponding to 10 % of the Swedish land area. Forest status was determined from satellite images and Swedish National Forest Inventory data using the probabilistic classifier method, which was used to model stream water chemistry with Bayesian model averaging. The results indicate that concentrations of e.g. nitrogen, phosphorus and organic matter are related to factors associated with forest production but that it is not forestry per se that causes the excess losses. Instead, factors simultaneously affecting forest production and stream water chemistry, such as climate, extensive soil pools and nitrogen deposition, are the most likely candidates The relationships with clear-felled and wetland areas are likely to be direct effects.

## Introduction

Effects of forest management on water quality are typically studied using plot- or catchment-scale experiments at sites as homogenous as possible and where most or all of the study area is treated. However, the Swedish boreal landscape is a mosaic of different land cover, and the spatial distribution of forestry operations on productive forestland is heterogeneous due to a multitude of factors. Productive forestland is the dominant (55 %) land cover in Sweden (land area 40.8 million hectares), while impediments such as rock surfaces, bog and marsh land, and mountains and alpine coniferous forest constitute another 2, 11 and 9 %, respectively. National parks, nature reserves and other protected areas cover an additional 10 % of Sweden and generally represent these types of land cover (Swedish Forest Agency 2012). Forest management activities are the main human impact on the productive forests, while the most important human impacts on impediment lands and protected areas are atmospheric deposition and presumably climate change.

While all forest management activities can impact surface water quality, the effects of final felling and subsequent site preparation are the most dramatic. Elevated nitrogen (N), phosphorus (P), dissolved organic carbon (DOC, Ahtiainen and Huttunen 1999; Akselsson et al. 2004; Löfgren 2007; Piirainen et al. 2007; Schelker et al. 2012) and base cation (Löfgren et al. 2009b; Piirainen et al. 2009; Rosén et al. 1996) fluxes to small headwater streams have been observed, sometimes up to more than a decade after final felling in the boreal zone of Fennoscandia and North America (Jerabkova et al. 2011; Kreutzweiser et al. 2008; Lamontagne et al. 2000). Nitrogen and phosphorus fertilization (Binkley et al. 1999; Lundin and Bergquist 1985) and drainage including ditch maintenance (Ahtiainen and Huttunen 1999; Joensuu et al. 2002; Lundin and Bergquist 1990; Nilsson and Lundin 1996) have effects on water quality for more than 5 years after treatment. Thinning and retention harvest seem to have less aquatic impact (Akselsson et al. 2004; Jerabkova et al. 2011), while differences in effects between tree species are largely unknown. Scots pine (*Pinus sylvestris* L.), Norway spruce (*Picea abies* (L.) Karst.) and birch (*Betula* spp.) are the three dominant tree species in Sweden making up more than 90 % of the standing biomass (Swedish Forest Agency 2012). Of these, Scots pine and Norway spruce are the most common species for regeneration and plantations, while planting exotic tree species is rare (Gustafsson et al. 2010).

Almost 90 million m^3^sk (stem volume over bark from stump to tip) is felled annually in Sweden. Final felling is annually performed on approximately 225,000 ha (ca 1 % of the productive forestland), of which ca 85 % is soil scarified and 75 % planted for regeneration. Pre-commercial thinning occurs on approximately 1.5 % of the productive forestland, while 2 % is commercially thinned and 0.3 % fertilized (Swedish Forest Agency 2012) with up to 200 kg N ha^−1^ (Swedish Forest Agency 2007). More than 1 million ha forestland on peat or wet mineral soils has been drained (Hånell and Magnusson 2005), and protective ditching is performed annually in around 1,000 ha (Swedish Forest Agency 2012). Except for harvesting, these measures are taken in order to stimulate forest production. Since the 1920s, the total standing volume of Swedish forests has increased by over 80 % (Swedish Forest Agency 2012).

At the landscape level, historical management determines current tree species composition and age structure of each stand. At the stand level, tree species composition and age are important parameters for identifying when a certain forestry operation should be undertaken. However, the timing and location of forest management activities are controlled by landowner decisions. These decisions are influenced by the market economy, legislation, Forest Stewardship Council (FSC) and Program for the Endorsement of Forest Certification (PEFC) certification rules, as well as consideration for other ecosystem services such as cultural heritage, biological diversity, hunting, etc. The consequences of these decisions and the effects of different ownership structures have a tangible effect on the forest landscape mosaic. In Sweden, private individuals own 50 % of the productive forestland, while big private and government owned companies dominate the other half. In 2011, e.g. the average notified areas for planned regeneration felling were 3.4 and 7.0 ha felling^−1^ for individual and other owners, respectively (Swedish Forest Agency 2012). This areal difference has a landscape-scale effect on subsequent forestry operations, potentially affecting water quality throughout the following rotation period.

Model results suggest that forestland contributes 40 % of nitrogen (N) and 35 % of phosphorus (P) net diffuse pollution from Sweden to the sea, of which final felling areas contribute 7 % (N) and 2 % (P) (Brandt et al. 2009; HELCOM 2011). Hence, at the national level, final felling is of minor importance for nutrient loadings to the sea. Locally, however, final felling may have substantial impacts on stream water quality. The density of perennial headwater streams is high, ca 1 km km^−2^, in the Swedish boreal landscape (Bishop et al. 2008; Ring et al. 2008). Due to this, boreal water bodies may fail to reach good ecological status according to the EU Water Framework Directive (Eriksson et al. 2011; Löfgren et al. 2009a), and local groundwater aquifers may be contaminated with nitrate (Futter et al. 2010).

From a policy point of view, it is important to understand forestry effects on surface waters from a landscape perspective. The EU Water Framework Directive (2000/60/EC), implemented in the Swedish Environmental Act, demands remedial actions for water bodies not achieving good ecological status. In Sweden, there are approximately 23,000 surface water bodies, of which 44 % had moderate ecological status or worse in 2009 (Swedish Water Authorities 2010). Many of these water bodies drain catchments with mixed land cover and a mosaic of managed forest stands. It is therefore important for the forestry sector and water authorities to be able to identify where, in the forested landscape, special precautions are necessary and where it can be assumed that forestry plays an important role for water quality.

In this study, we take this landscape approach and use water chemical data from almost 200 randomly selected headwater streams in the hemiboreal zone (southern boreal and boreonemoral) of southwest and central Sweden for assessing the impact of forest and wetland status on stream water quality. The subsample of streams represents hemiboreal headwaters within an area of 43,000 km^2^, which equals 10 % of the total land area of Sweden. Using data from the Swedish National Forest Inventory (NFI), satellite images and the probabilistic classifier method, the relations between typical forestry parameters (basal area, biomass, increment, tree species composition, clear-felled area) and stream water concentrations of nutrients, organic matter and ions affecting acid-base chemistry were quantified. The results were assessed in the context of different climate, sulphur and nitrogen deposition, as well as marine influences.

## Material and methods

### Stream selection

**Fig. 1:**
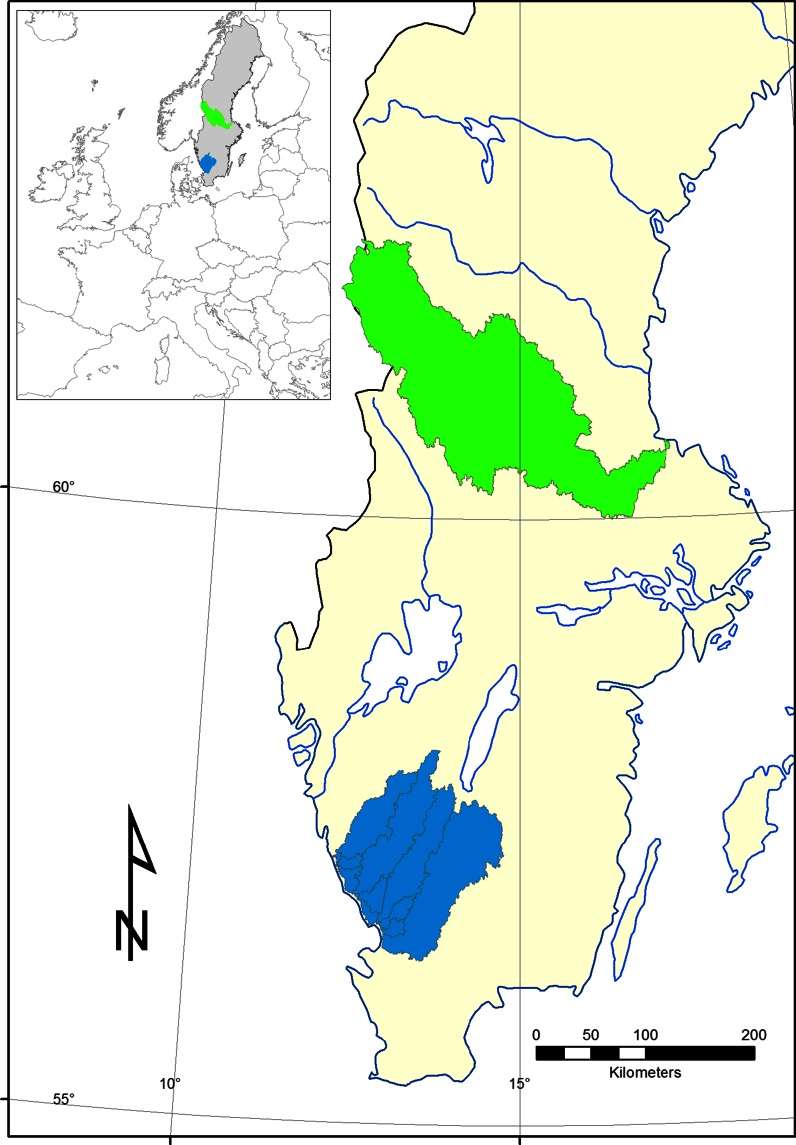
Main catchments with randomly selected headwater catchments. *Green* Dalälven catchment, *blue* southwest region (Viskan, Ätran, Nissan and Lagan catchments)

In each of two different regions of Sweden, 100 first-order headwater streams were selected randomly. In central Sweden, the selected headwater subcatchments were located within the river Dalälven catchment. In southwest, the headwater catchments were located within the catchments of four major rivers: Viskan, Ätran, Nissan and Lagan (Fig. 1). The headwater streams were identified based on a ‘virtual hydrological network’ generated from a 50 m × 50 m digital elevation model (Nisell et al. 2007). The object-oriented database contains water bodies, watersheds and their topological relations. The datasets contain raster data on flow accumulation and flow direction as well as vector data on river reaches, lakes and contributing areas on a national scale. The database includes 930,000 stream reaches/arcs, and in this study, headwaters are defined as arcs with a start node without discharge from another upstream arc. The threshold value for accumulated flow, defining the initiation of a headwater stream in the landscape, varied between 1.1 and 3.5 l s^−1^ across Sweden.

The water divide for each headwater catchment was modelled using the same elevation data, while land cover and roads were mapped from digital versions of the topographic map (1:50,000) and road map (1:100,000), respectively. Metria (http://www.metria.se/Startpage/) provided all geographical data except the ‘virtual network’. One hundred headwaters fulfilling the criteria of, being longer than 2,500 m (to ensure that the stream was not ephemeral), having a distance of <500 m to a driveable road at the outlet, without lakes and urban areas and with <5 % agricultural land in the catchment, were randomly selected in each region. Later quality control, based on land cover data classified from satellite images (25 m × 25 m pixel, GSD-Marktäckedata, Metria) and the county administrations’ surface water liming register, showed that some catchments were not suitable and therefore excluded as they either were limed, contained some urban area or had more than 5 % agricultural land.

The small shares of agricultural land in these forested catchments (≤4.9 %) are non-fertilized grasslands used for extensive grazing and with nutrient losses similar to those found from forests (Brandt et al. 2009). Based on experimental data from field trials, production region, crop rotation, fertilizer levels, soil texture, harvest and climate, the nitrogen losses from grazing land have been estimated by model simulations (HBV-N, Brandt and Ejhed 2008). These simulations indicate an average nitrogen concentration of 0.5 mg N/L in Dalälven and 0.7 mg N/L in the southwest. Based on the mean nitrogen concentrations 0.41 and 0.85 mg N L^−1^, respectively, in this investigation, the share of grazing land must be >55 % in order to increase the nitrogen concentration with more than 10 % in Dalälven. Thus, the low number of catchments (40 out of 179) with small shares of agricultural land (≤4.9 %) does not tangibly affect the models. It is the same with the low share of water surface area. Analysis of GSD-Marktäckedata showed that 21 catchments included surface water pixels representing the stream itself as well as tarns, ponds, etc. which lacked visible connectivity to the stream. In 19 of the catchments, the water surface area was <1 %, while in 4 catchments the range was 1–5 %. All these catchments were accepted. Therefore, the final number of catchments was 84 in the southwest and 95 in the Dalälven region. Due to the length of a tree generation (>50 years), average climate data for the period 1961–1990 were used and obtained from the Swedish Meteorological and Hydrological Institute (SMHI) (Raab and Vedin 1995). We excluded grazing land and water surface area from the models reported here.

### Water sampling and chemical analyses

Water was sampled during four different seasons (Table 1). Except for the summer sampling in Dalälven 2009, which was conducted by Swedish University of Agricultural Sciences (SLU) staff, the sampling was performed by the county administration boards in Dalarna, Västra Götaland, Jönköping and Halland. The collected water was sent to the laboratory at the Department of Aquatic Sciences and Assessment, SLU, for chemical analyses, which were initiated within 1 day of sampling.

**Table 1:** Sampling dates in the headwater streams in the catchments of Dalälven and Viskan, Ätran, Nissan and Lagan (southwest)

	Dalälven	Southwest
Summer	August 10–September 2, 2009	May 31–June 7, 2010
Spring	May 2–5, 2011	April 4–7, 2011
Autumn	September 12–15, 2011	September 12–15, 2011
Late autumn	November 7–11, 2011	November 28–December 1, 2011

The analytical methods are accredited by the Swedish Board for Accreditation and Conformity Assessment (www.swedac.se) and follow the Swedish standard methods. pH was measured in a through-flow cuvette using a Radiometer PHM 210 Precision pH meter at ambient pCO_2_ pressure (pH). Total organic carbon (TOC) was measured using a Shimatzu TOC 5050 analyzer with ASI-502 sample injector following acidification. Analysis of calcium (Ca^2+^), magnesium (Mg^2+^), sodium (Na^+^) and potassium (K^+^) was performed by inductively coupled plasma atomic emission spectroscopy (ICP-AES) (Varian Vista Ax Pro) and of sulphate (SO4^2−^) and chloride (Cl^−^) by ion chromatography (LDC ConductoMonitor III). Ammonium (NH_4_^+^, indofenol method), nitrate (NO_3_^−^, sulphanilamide method after Cd reduction), total phosphorus (TP, molybdenum method after persulphate digestion) and phosphate (PO_4_^3−^, molybdenum method) were photometrically analyzed (Bran Luebbe Autoanalyzer 3). Total nitrogen (TN) was measured using a TNMI-module equipped Shimatzu TOC-VCPH analyzer. Iron (Fe) and manganese (Mn) were analyzed with ICP-AES (Varian Vista AX Pro). Further information on the analytical methods, including analytical precision and limits of detection, can be found at the department’s website (SLU 2014a). Except for NO_3_^−^, all observations were above the limits of detection. The latter is 1 μg L^−1^ for NO_3_^−^, which was used as NO_3_^−^ concentration on samples below this limit. Acid-neutralizing capacity (ANC, μeq L^−1^) is calculated according to Reuss and Johnson (1986):

1 $$ \mathrm{A}\mathrm{N}\mathrm{C}={\mathrm{Ca}}^{2+}+{\mathrm{Mg}}^{2+}+{\mathrm{K}}^{+}+{\mathrm{Na}}^{+}-\left({{\mathrm{SO}}_4}^{2-}+{{\mathrm{NO}}_3}^{-}+{\mathrm{Cl}}^{-}\right)\left(\mu \mathrm{eq}{\mathrm{L}}^{-1}\right) $$

### Forest and wetland status classification

The status of forests and wetlands was classified using the non-parametric probabilistic classifier method (Yu and Ranneby 2007a; b) based on remote sensing data (LandsatTM and Spot in Dalälven and southwest, respectively), forest and wetland data from the Swedish NFI (tree species composition, tree age, tree biomass, average increment, basal area etc.) and final felled areas (Swedish Forest Agency and Metria). The NFI data was from 2005 to 2009, representing 5-year average tree growth and tree biomass excluding other vegetation (SLU 2014b). The classification of deciduous trees was improved by using satellite images (AWifs) representing time periods after leaf fall. At the pixel level, seven forest and two wetland variables (Table 2) were quantified and aggregated to catchment area. The portion of deciduous trees was classified before conifer classes were determined. The pixel size was 25 × 25 m in Dalälven and 10 × 10 m in the southwest. Accumulated final felled area during the last 10 years, based on polygons obtained from the Swedish Forest Agency, constituted the eighth forest class. In northern Sweden, the general view is that final felling affects N and P concentrations for 10–15 years, while the same figure is around 5 years in southern Sweden (Löfgren 2007 and references therein). A period of 10 years has been used for both regions, due to the restricted number of studies supporting these generalizations. Both drained and non-drained forests are most probably represented in the selected catchments, and potential effects on water chemistry are likely in the studied streams. However, it is not possible to add drainage as a spatial parameter in the models since no data are available on where and to what extent the forests are drained. For the same reason, fertilization or ash return is not included as a parameter in the models. The probabilistic classifier method derives unbiased area estimates for small areas and deviating areas based on information about the probability distribution at pixel level (Yu and Ranneby 2007a, b).

**Table 2:** Probabilistic classifier method derived forest and wetland classes (variables and units) used for the BMA modelling

Land cover	Name	Variable	Unit
Forest	Growth	Average increment	m^3^ ha^−1^ year^−1^
	Biomass	Average total biomass	Kton ha^−1^
	>70 % spruce	Norway spruce ≥70 %	share of catchment %
	>70 % pine	Scots pine ≥70 %	share of catchment %
	>50 % deciduous	Deciduous trees >50 %	share of catchment %
	20–50 % deciduous	Deciduous trees [20 %, 50 %)	share of catchment %
	>70 % mixed conifers	Mixed conifers ≥70 %	share of catchment %
	Clear-cut	Final felled area	share of catchment %
Wetland	Wetland forested	Basal area ≤3 m^2^ ha^−1^	share of catchment %
	Wetland non-forest	Basal area >3 m^2^ ha^−1^	share of catchment %

Slowly weathering granite, gneiss, porphyry and sandstone are the most abundant minerals in the studied catchments (Swedish Geological Survey, data not shown). Except for two catchments in the central part of the Dalälven region (1 and 13 % of the catchment area, respectively), calcium-rich soils (calcite, dolomite, etc.) do not occur in any of the catchments. Thus, the impact of calcium-rich soils is negligible in all catchments except one, and including this as a parameter would not add any extra information to the models. Variations in lithology, mineral composition and soil chemistry between catchments are taken into account by the random sampling, which has a consequence of reducing the explanatory power by the models.

### Stream water chemistry models

The relations between forest and wetland status and stream water chemistry were studied using Bayesian model averaging (BMA) (Feldkircher and Zeugner 2009). For each chemical constituent (log_10_ transformed), models were created with landscape parameters (Table 2) as explanatory variables. An advantage of the BMA method is that effect sizes are not calculated from one single model but instead calculated based on all considered models (in this case, 1,024), weighted by the posterior model probabilities. Thus, this method takes model uncertainty into account. The posterior inclusion probability for each explanatory variable is also estimated. This is a measure of how important this variable is for explaining observations and the fraction of positive coefficients, conditional on inclusion.

The BMA analyses were performed using R 3.00 and the package ‘BMS’. In the BMA analysis, all possible models were run and evaluated, and uniform model priors were used. The performance of the models was assessed by 10-fold cross-validation.

The explanatory variables in the models are assumed to be constant over the short-term (decadal) time scale and related to spatial variations in forest and wetland properties (Table 2). Climatic and other temporal parameters were not used in the models. In order to test whether there were seasonal differences in explanatory power, models were created based on water chemistry from four different seasons. Both seasonal and regional (Dalälven and southwest) models were used to interpret possible mechanisms behind the variation in water chemistry.

## Results

### Catchment characteristics

A summary of catchment characteristics for the studied first-order streams is presented in Table 3. There are tangible differences in catchment properties between the two regions. Based on the non-parametric Wilcoxon test (JMP 9.0.0, *p* < 0.05), the catchments in the Dalälven region were larger, located at higher altitude with lower mean annual temperatures and precipitation and a higher proportion of Scots pine compared with catchments in the southwest (Table 3). In the latter region, atmospheric deposition of sulphate-sulphur (S), inorganic nitrogen (N) and chloride (Cl^−^); forest growth; forest biomass; the proportion of Norway spruce and proportion of clear-cuts were all higher (Table 3). Historically, the difference in S deposition between the regions has been even larger (Westling and Lövblad 2000). The forest growth figures in Table 3 may seem low for both regions, but they represent average production for the entire catchment including both productive forestland and impediments such as wetlands, bare rock, etc.

**Table 3:** Catchment properties in the two regions Dalälven and southwest

	Dalälven						Southwest					
	Min	25 (%)	Median	75 (%)	Max	Mean	Min	25 (%)	Median	75 (%)	Max	Mean
Catchment area (ha)	106	163	209	252	620	221	24	79	105	149	279	117
Precipitation (mm year^−1^)	650	750	750	850	950	780	750	950	950	1,050	1,250	996
MAT (°C)	0.5	1.5	2.5	3.5	4.5	2.6	4.5	5.5	5.5	6.5	6.5	5.9
Vegetation period (days)	140	160	160	170	180	164	190	190	200	200	210	196
NH_4_-N deposition (kg ha^−1^ year^−1^)	1.1	1.3	1.5	1.6	2.1	1.5	3.5	4.6	5.4	6.2	8.0	5.4
NO_3_-N deposition (kg ha^−1^ year^−1^)	1.8	2.3	2.4	2.5	3.0	2.4	4.3	5.1	5.4	5.5	5.8	5.3
SO_4_-S deposition (kg ha^−1^ year^−1^)	1.3	1.8	1.9	2.0	2.8	1.9	3.8	4.6	4.9	5.1	5.7	4.8
Cl deposition^a^ (kg ha^−1^ year^−1^)	2	–	–	–	7	–	10	–	–	–	50	–
Forest growth (m^3^ ha^−1^ year^−1^)	0.8	1.8	2.3	2.9	4.1	2.3	0.8	3.5	4.3	5.0	7.0	4.2
Forest biomass (kton ha^−1^)	30	52	65	83	110	68	22	84	97	109	146	95
Wetland forested (%)	0.3	8.2	11.8	17.3	41.2	12.9	3.7	10.6	14.4	18.9	36.0	15.0
Wetland non-forest (%)	0.2	5.9	9.1	14.8	32.3	10.5	0.6	2.8	4.6	11.3	64.7	9.1
Clear-cut (%)	0.0	2.5	8.3	13.5	45.7	9.8	0.0	6.7	10.9	17.9	55.8	12.9
>70 % spruce (%)	1.1	6.1	9.5	14.6	29.7	10.8	1.3	15.5	22.7	28.5	63.3	22.9
>70 % pine (%)	6.4	16.9	23.5	33.7	65.7	25.7	2.3	7.5	11.1	14.7	27.3	11.4
>50 % deciduous (%)	0.7	4.1	5.5	7.0	13.3	5.7	0.5	2.3	3.6	6.9	23.3	5.1
20–50 % deciduous (%)	4.1	11.4	14.7	18.7	33.5	15.4	3.8	9.7	11.6	14.3	24.3	12.3
>70 % mixed conifers (%)	1.6	5.5	8.4	10.5	18.4	8.4	1.2	6.4	8.9	11.3	27.0	9.2
Agricultural land (%)	0	0	0	0	2.4	0.1	0	0	0.2	2.1	4.9	1.2

^a^Throughfall data 1995–2012 from the Swedish Throughfall Monitoring Network (http://www.krondroppsnatet.ivl.se/)

### Stream water chemistry—regional differences

The concentrations for most studied elements were, on average, significantly (Wilcoxon test, *p* < 0.05) higher in the southwest than in the Dalälven region (Table 4). Concentrations of TN and TP were generally twice as high in the southwest compared with Dalälven. In both regions, organically bound nitrogen (OrgN) dominated, reflecting the differences in TOC concentrations with tangibly higher levels in the southwest. However, the proportion of inorganic nitrogen (TIN), especially NO_3_^−^, was at least 3-fold higher in the southwest. The most pronounced difference was for Cl^−^, which was, on average, more than five times higher than the concentrations in the southwest, followed by Na^+^, which had a 3-fold difference or more between the two regions. Also, SO_4_^2−^ concentrations were at least twice as high in the southwest. The mobile mineral acid anions SO_4_^2−^, Cl^−^ and NO_3_^−^ were in large excess in the southwest compared with Dalälven. However, for the balancing base cation Ca^2+^, the difference in concentrations between regions was small, with higher average concentrations in Dalälven during all times of the year except summer. This was in contrast to Mg^2+^ and K^+^, which followed the general pattern of higher concentrations in the southwest. Dalälven streams had much lower concentrations of strong mineral acid anions. With the exception of spring snowmelt, this was reflected in well-buffered conditions with tangibly higher ANC and pH compared to streams in the southwest. The generally higher strong mineral acid anion concentrations in the southwest are in accordance with other independent data from national surface water monitoring programs (Löfgren et al. 2010b; Wilander et al. 2003).

**Table 4:** Concentrations in randomly selected headwater streams in A the River Dalälven catchment and B the southwest region during different seasons (see Table 1

	Spring					Summer					Autumn					Late autumn				
	Min	25 (%)	Median	75 (%)	Max	Min	25 (%)	Median	75 (%)	Max	Min	25 (%)	Median	75 (%)	Max	Min	25 (%)	Median	75 (%)	Max
A	Dalälven																			
TOC (mg L^−1^)	2.5	6.6	8.8	12.3	27.9	2.6	15.4	22.0	27.8	58.3	3.8	17.3	22.4	27.8	46.7	1.8	9.5	14.8	20.2	37.5
TN (μg L^−1^)	85	187	238	304	1,108	62	313	453	596	2,050	94	340	435	555	1,076	64	215	288	413	849
OrgN (μg L^−1^)	79	166	206	265	593	56	300	422	562	1,779	92	333	414	517	930	35	177	249	344	733
NO_3_-N (μg L^−1^)	1	6	19	53	951	1	3	4	10	719	1	3	4	8	205	4	12	25	50	249
NH_4_-N (μg L^−1^)	1	1	1	4	65	3	6	8	11	255	1	3	5	9	41	1	4	8	19	194
TIN (μg L^−1^)	2	7	20	63	956	4	10	15	21	727	2	6	12	19	213	6	20	41	72	277
TP (μg L^−1^)	1	4	6	8	79	2	8	11	16	44	2	7	11	14	37	1	5	8	12	82
ResP (μg L^−1^)	0	1	3	4	74	0	5	7	11	39	0	4	7	11	27	0	2	4	7	79
PO_4_-P (μg L^−1^)	1	2	3	4	14	1	3	4	5	21	1	3	3	4	20	1	3	4	5	17
pH	4.9	5.8	6.2	6.6	6.9	4.3	5.1	5.8	6.3	7.1	4.2	4.9	5.7	6.2	6.9	4.6	5.9	6.3	6.6	7.2
ANC (μeq L^−1^)	29	90	124	191	440	57	134	179	241	560	67	138	176	235	656	51	137	174	221	666
Ca^2+^ (μeq L^−1^)	15	61	87	139	371	26	87	128	166	466	37	89	121	160	529	24	87	121	161	519
Mg^2+^ (μeq L^−1^)	8	22	32	50	133	11	28	41	60	158	12	29	39	57	154	12	32	41	58	192
Na^+^ (μeq L^−1^)	24	41	52	64	293	23	36	52	69	380	23	37	49	63	182	24	43	54	74	278
K^+^ (μeq L^−1^)	1	6	8	10	16	0	3	5	7	16	3	7	9	11	18	1	6	8	11	18
SO_4_^2−^ (μeq L^−1^)	8	22	29	45	133	4	9	16	27	90	5	10	14	29	167	7	15	21	32	124
Cl^−^ (μeq L^−1^)	6	11	15	23	312	7	14	21	31	407	11	16	22	33	124	7	16	22	35	326
NO_3_^−^ (μeq L^−1^)	1	1	1	4	68	1	1	1	1	51	1	1	1	1	15	1	1	2	4	18
Fe (μg L^−1^)	10	243	460	683	2,800	10	650	1,100	1,850	14,000	24	670	1,050	1,700	3,700	11	613	970	1,800	5,200
Mn (μg L^−1^)	1	11	21	41	870	1	26	43	97	4,400	1	26	48	94	1,300	2	20	45	110	1,200
B	Southwest																			
TOC (mg L^−1^)	6.3	14.7	18.2	22.9	30.9	8.0	20.6	25.6	35.2	51.7	12.3	34.7	43.0	52.3	77.0	8.6	20.9	28.8	35.6	67.6
TN (μg L^−1^)	166	511	645	837	1,857	259	590	759	967	2,013	393	787	1,010	1,260	2,833	377	566	763	970	2,401
OrgN (μg L^−1^)	149	357	464	544	927	236	490	615	789	1,729	305	764	958	1,174	1,983	256	470	586	783	1,555
NO_3_ (μg L^−1^)	5	83	134	229	557	2	27	55	106	616	4	9	23	48	135	5	37	73	128	483
NH_4_ (μg L^−1^)	1	16	39	84	775	2	13	28	74	569	3	10	17	33	799	4	21	44	79	1,054
TIN (μg L^−1^)	7	117	187	319	949	9	48	117	217	718	7	20	44	86	850	10	71	136	199	1,190
TP (μg L^−1^)	5	10	13	18	50	5	13	17	24	66	8	16	21	30	63	5	11	15	23	52
ResP (μg L^−1^)	2	7	10	13	45	3	7	10	15	44	4	12	17	22	44	2	7	10	15	31
PO_4_ (μg L^−1^)	1	2	3	5	9	2	5	6	9	32	2	3	5	7	20	2	4	5	7	21
pH	4.2	4.6	4.8	5.0	6.8	4.4	5.0	5.4	6.1	7.5	4.0	4.4	4.6	5.0	6.9	4.0	4.4	4.7	5.0	6.9
ANC (μeq L^−1^)	−11	26	48	82	297	27	97	159	277	1,199	42	105	136	243	756	−17	40	73	123	487
Ca (μeq L^−1^)	16	42	59	84	302	33	80	120	200	1,112	26	63	82	138	700	31	61	89	117	506
Mg (μeq L^−1^)	16	35	45	58	103	36	64	82	103	259	31	53	68	85	135	42	58	71	88	118
Na (μeq L^−1^)	66	112	142	162	1,495	120	176	193	223	793	81	138	160	183	534	107	164	192	216	675
K (μeq L^−1^)	7	13	15	18	37	1	8	11	16	48	3	7	9	13	24	5	12	15	18	27
SO_4_ (μeq L^−1^)	18	53	65	81	165	8	37	54	81	160	6	28	36	47	109	23	48	56	70	140
Cl (μeq L^−1^)	47	97	129	158	1,575	92	146	170	212	802	69	118	144	160	465	106	193	222	251	717
NO_3_ (μeq L^−1^)	1	6	10	16	40	1	2	4	8	44	1	1	2	3	10	1	3	5	9	35
Fe (μg L^−1^)	10	795	1,100	1,500	3,500	130	1,675	2,450	3,425	12,000	890	2,325	3,000	4,550	14,000	640	1,500	2,000	2,850	11,000
Mn (μg L^−1^)	16	42	59	85	380	12	48	69	103	1,100	19	51	77	120	2,200	20	51	73	110	600

25 %, 25th percentile; 75 %, 75th percentile

### Stream water chemistry—seasonal differences

There were significant differences (Wilcoxon test, *p* < 0.05, JMP 9.0.0) between seasons for most water chemical variables, except for between summer and autumn in Dalälven and between summer and late autumn in the southwest (Table 4). The variables associated with TOC, i.e. primarily dissolved organic matter, but also TN and TP, had low concentrations in spring and late autumn and high concentrations during summer and autumn. Similarly, the base cations, with the exception of K^+^, had the lowest concentrations in the spring. Highest base cation concentrations, again with the exception of K^+^, were recorded during summer. In contrast, SO_4_^2−^ and TIN, especially NO_3_^−^, had the highest concentrations in spring and the lowest concentrations in autumn (Table 4). Due to the seasonality in ion composition, ANC was highest in summer. This was reflected also in the high pH in southwest streams. In contrast, pH was highest in spring and late autumn in Dalälven.

During summer, the concentration differences in TOC, TN, TP and pH between regions were generally smaller than during the other seasons (Table 4). The relatively similar concentrations between regions coincided with low coefficient of determination for seasonal models of TOC, TN, TP and pH for the summer period (Table 5).

**Table 5:** Coefficient of determination (*r* ^2^ values) for the best models based on seasonal data from both regions and separated for Dalälven and southwest

Constituent	Both regions					Dalälven					Southwest				
	Spring	Summer	Autumn	Late autumn	All seasons	Spring	Summer	Autumn	Late autumn	All seasons	Spring	Summer	Autumn	Late autumn	All seasons
TOC	0.55	0.25	0.52	0.51	0.46	0.49	0.47	0.31	0.46	0.43	0.21	0.27	0.24	0.20	0.23
TN	0.66	0.46	0.61	0.63	0.59	0.48	0.52	0.42	0.57	0.50	0.16	0.28	0.15	0.07	0.17
NO_3_-N	0.57	0.49	0.38	0.37	0.45	0.50	0.26	0.29	0.40	0.36	0.24	0.04	0.08	0.11	0.12
NH_4_-N	0.64	0.47	0.40	0.46	0.49	0.30	0.22	0.11	0.41	0.26	0.30	0.37	0.18	0.09	0.23
TIN	0.59	0.48	0.37	0.40	0.46	0.49	0.22	0.31	0.40	0.35	0.15	0.11	0.05	0.05	0.09
TP	0.37	0.29	0.35	0.25	0.32	0.19	0.38	0.19	0.13	0.22	0.06	0.13	0.05	0.05	0.07
ResP	0.38	0.29	0.33	0.28	0.32	0.15	0.41	0.17	0.18	0.23	0.05	0.09	0.06	0.05	0.06
PO_4_-P	0.03	0.24	0.15	0.04	0.46	0.20	0.11	0.13	0.02	0.46	0.06	0.12	0.04	0.04	0.46
pH	0.54	0.13	0.35	0.49	0.38	0.18	0.22	0.18	0.17	0.19	0.25	0.27	0.18	0.18	0.22
ANC	0.20	0.06	0.10	0.13	0.12	0.22	0.40	0.43	0.27	0.33	0.29	0.25	0.06	0.06	0.17
Ca^2+^	0.15	0.17	0.14	0.12	0.14	0.35	0.49	0.52	0.35	0.43	0.07	0.25	0.05	0.07	0.11
Mg^2+^	0.35	0.45	0.43	0.45	0.42	0.37	0.41	0.46	0.34	0.40	0.31	0.14	0.09	0.16	0.17
Na^+^	0.72	0.77	0.78	0.78	0.76	0.48	0.50	0.54	0.51	0.51	0.45	0.32	0.48	0.58	0.46
K^+^	0.41	0.39	0.06	0.37	0.31	0.24	0.35	0.29	0.27	0.29	0.22	0.10	0.07	0.39	0.19
SO_4_^2−^	0.64	0.61	0.54	0.59	0.59	0.54	0.50	0.44	0.41	0.47	0.65	0.32	0.50	0.53	0.50
Cl^−^	0.80	0.79	0.82	0.82	0.81	0.49	0.38	0.51	0.57	0.49	0.60	0.42	0.48	0.50	0.50
Fe	0.42	0.42	0.60	0.48	0.48	0.48	0.48	0.47	0.50	0.48	0.12	0.35	0.30	0.36	0.28
Mn	0.40	0.18	0.25	0.20	0.26	0.37	0.32	0.42	0.38	0.37	0.28	0.03	0.38	0.24	0.23

### Landscape and stream water chemistry relations

**Fig. 2:**
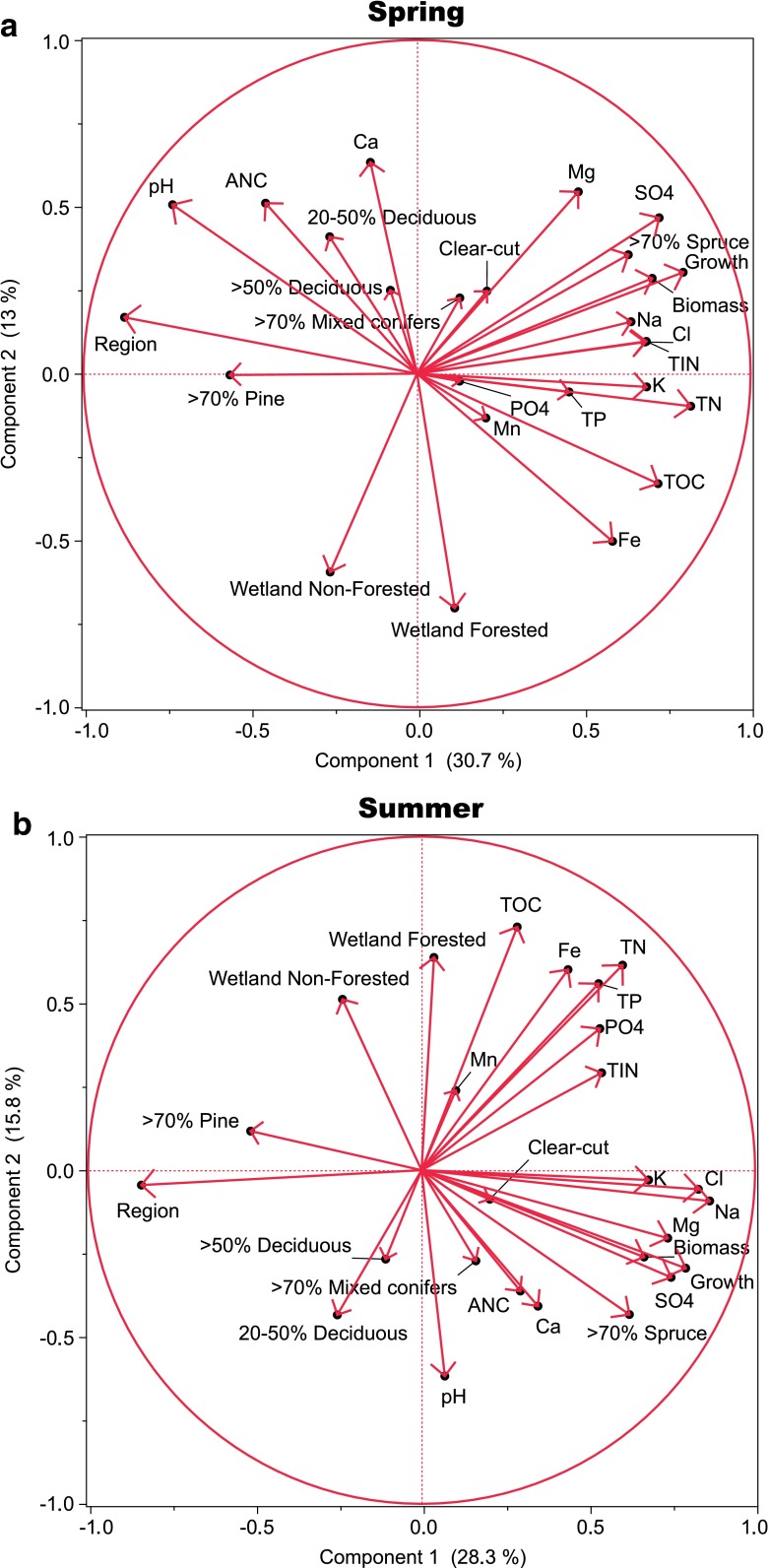
Co-variations between modelled parameters in spring and summer, which are the seasons when the models had the highest and lowest coefficients of determinations, respectively (Table 5). Loading plots (principal component analysis)

Figure 2 shows the co-variation between forest and water chemistry parameters in spring and summer, which are the seasons when the models had highest and lowest coefficients of determination, respectively (Table 5). In both cases, the first two components (loading plots, principal component analysis, JMP 10.0) explain 44 % of the variation in the dataset. The first component represents regional differences, while the second component represents gradients related to the proportion of wetlands in the catchments. The proportions of deciduous trees, mixed conifers and clear-fellings were not related to any of these gradients, while forest production, biomass, Norway spruce and Scots pine were related to the regional gradient. The proportions of mixed conifers and deciduous trees were related to principal components 3 and 4, respectively, explaining 11 and 7 % of the variation. In spring, the chemical constituents were also related to this regional gradient except for Ca^2+^, which was related to the wetland gradient, and PO_4_^3−^ and Mn, which were unrelated to either component. In summer, the chemical constituents arranged somewhat differently, and the wetland gradient became more important for pH, TOC, TN, TP and Fe (Fig. 2).

The three best predicted chemical variables were Na^+^, Cl^−^ and SO_4_^2−^ (0.5 ≤ *r* ^2^ ≤ 0.8, Table 5). These variables were all well predicted both in the combined modelling of both regions and in the separate regional models (Table 5). Total nitrogen was also relatively well predicted (0.5 ≤ *r* ^2^ ≤ 0.7, Table 5), generally better than TOC and TIN (0.3 ≤ *r* ^2^ ≤ 0.6 and 0.3 ≤ *r* ^2^ ≤ 0.4, respectively), but the regional TN models were less precise, especially in the southwest (*r* ^2^_Southwest_ < 0.28, *r* ^2^_Dalälven_ < 0.57). Typically, for most variables, explanatory power was not as good for the regional models as for the main models (Table 5).

In general, forest growth was the most important explanatory variable in the models. Forest growth occurred in nearly all of the top models as a positive factor for concentrations of the chemical species (Table 6a).

**Table 6:** (A) Regression coefficients for the best models based on seasonal data from both regions (Dalälven and southwest). (B) Regression coefficients for the best models based on both regions and all seasons

A	Response variables																	
	TOC				TN				NO_3_-N				NH_4_-N					
	1	2	3	4	1	2	3	4	1	2	3	4	1	2	3	4		
Predictors (catchment properties)																		
Intercept	1.03	1.06	1.30	0.40	2.81	2.06	2.64	1.65	0.54	0.43	0.37	0.20	0.54	0.24	0.22	0.28		
Growth	0.13	0.06	0.12	0.14	0.19	0.15	0.17	0.17	−0.67	1.28	0.58	0.98	1.55	−0.19	−0.49	−0.46		
Biomass																		
Wetland forested	0.89	1.30	1.10	1.99		1.20	0.81	1.75						2.38	3.16	3.17		
Wetland non-forest				0.82		0.92		0.97	2.66					2.89	1.88	1.85		
Clear-cut				0.62		0.60		1.02	3.02			1.39		1.05	1.13	1.52		
>70 % spruce	−1.06	−0.54	−0.92		−1.63	−0.64	−1.36		−1.95	−3.61	−2.93		−4.58					
>70 % pine	−0.89		−0.76		−1.33		−1.01			−2.69	−1.45		−3.67					
>50 % deciduous			−1.35		−1.02		−1.34						−5.34	−2.25				
20–50 % deciduous	−0.98				−1.11				1.85	−2.68			−2.62					
>70 % mixed conifers					−1.18							−2.09	−3.23					
	Response variables																	
	TIN				TP				ResP				PO_4_-P					
	1	2	3	4	1	2	3	4	1	2	3	4	1	2	3	4		
Predictors (catchment properties)																		
Intercept	0.25	−0.26	1.43	1.30	1.21	1.31	0.54	0.40	0.23	0.08	0.12	0.18		0.12	0.11	0.04		
Growth	0.53	0.32	0.29	0.28	0.13	0.12	0.11	0.10	1.06	0.55	0.48	−0.29	0.61	0.77	0.40	0.54		
Biomass																		
Wetland forested		1.88					1.43	1.67		1.97	1.69	3.07						
Wetland non-forest	2.01	3.02																
Clear-cut	2.21	2.20		0.87			0.60	0.58										
>70 % spruce	−2.78		−2.55	−2.02	−1.22	−1.34			−1.91					−1.28	−0.90			
>70 % pine	−1.07		−1.64	−0.89	−1.03	−0.69			−1.90		−0.75			−0.69				
>50 % deciduous										2.80				−2.32				
20–50 % deciduous			−1.77		−2.05	−1.61			−3.65	−2.67								
>70 % mixed conifers													−0.90					
	Response variables																	
	pH				ANC				Cl^−^				SO_4_^2−^					
	1	2	3	4	1	2	3	4	1	2	3	4	1	2	3	4		
Predictors (catchment properties)																		
Intercept	6.59		5.57	6.05	0.21	0.22	0.21	0.28	−1.41	−1.26	−1.27	−1.10	−1.80	−2.17	−2.12	−1.95		
Growth	−0.33			−0.30		0.07	0.04		0.40	0.40	0.34	0.42	0.20	0.30	0.23	0.22		
Biomass			0.00			0.00							0.00	0.00	0.00	0.00		
Wetland forested	−4.81		−5.37	−5.52	−0.42		−0.44	−0.58										
Wetland non-forest																		
Clear-cut													0.98	1.45	1.20	1.03		
>70 % spruce					−0.29		−0.45	−0.27	−2.55	−2.60	−2.26	−2.86						
>70 % pine	2.79		2.80	3.70					−1.95	−2.17	−1.85	−2.38						
>50 % deciduous									−2.13	−2.24	−1.69	−2.19						
20–50 % deciduous	4.48		5.67	6.37					−1.41	−1.36	−1.21	−1.74						
>70 % mixed conifers									−1.58	−1.28	−1.13	−1.61						
	Response variables																	
	Ca^2+^				Mg^2+^				Na^+^				K^+^					
	1	2	3	4	1	2	3	4	1	2	3	4	1	2	3	4		
Predictors (catchment properties)																		
Intercept	−0.74	−0.79	−0.87	−0.67	−1.67	−1.89	−1.48	−1.47	−1.30	−1.58	−1.39	−1.48	−2.48	−2.78	−1.95	−2.42		
Growth		0.12	0.09		0.08	0.14	0.12	0.11	0.21	0.28	0.24	0.25	0.13	0.15		0.14		
Biomass																		
Wetland forested	−1.55	−1.32	−1.28	−1.43											−0.75			
Wetland non-forest						0.98				0.64		0.52	0.70	0.80				
Clear-cut					0.68	0.99	0.52	0.57	0.47	0.87	0.61	0.77	0.77	1.22		0.76		
>70 % spruce		−0.94	−1.24				−0.88	−0.63	−1.19	−1.39	−1.53	−1.33	−0.48			−0.67		
>70 % pine							−0.55	−0.55	−0.82	−0.93	−0.95	−0.89						
>50 % deciduous									−1.37	−1.22	−1.27	−1.10		−1.87				
20–50 % deciduous																		
>70 % mixed conifers	−2.06	−1.62		−1.36	−1.04				−0.78									
	Response variables																	
	Fe				Mn													
	1	2	3	4	1	2	3	4										
Predictors (catchment properties)																		
Intercept	1.51	2.08	2.63	2.28	0.04	1.12	0.86	0.87										
Growth	0.19	0.16	0.26	0.13	0.23	0.09	0.13	0.13										
Biomass																		
Wetland forested	3.19	3.16	2.82	3.70	2.80	2.53	2.57	2.50										
Wetland non-forest	1.26	1.34			1.15													
Clear-cut	0.75				1.06													
>70 % spruce			−1.77															
>70 % pine			−1.29	−0.54														
>50 % deciduous			−1.69		2.91		2.58	2.40										
20–50 % deciduous																		
>70 % mixed conifers																		
B	Response variables																	
	TOC	TN	NO_3_-N	NH_4_-N	TIN	TP	ResP	PO_4_-P	pH	ANC	Cl^−^	SO_4_^2−^	Ca^2+^	Mg^2+^	Na^+^	K^+^	Fe	Mn
Predictors (catchment properties)																		
Growth	0.11	0.17	0.39	0.31	0.35	0.11	0.16	0.06	−0.22	0.01	0.39	0.23	0.06	0.11	0.24	0.11	0.18	0.14
Biomass	0.00	0.00	0.00	0.00	0.00	0.00	0.00	0.00	0.00	0.00	0.00	0.00	0.00	0.00	0.00	0.00	0.00	0.00
Wetland forested	1.28	0.83	−0.06	2.24	0.44	0.79	1.63	0.14	−5.33	−0.27	0.15	−0.02	−1.30	0.01	0.08	−0.11	3.16	2.45
Wetland non-forest	0.18	0.38	0.69	1.70	1.30	0.02	0.11	0.08	−0.15	0.01	0.31	0.02	0.00	0.31	0.25	0.26	0.54	0.10
Clear-cut	0.12	0.32	1.26	0.93	1.30	0.20	0.10	0.16	−0.04	0.02	0.38	1.17	0.00	0.72	0.68	0.70	0.13	0.10
>70 % spruce	−0.62	−1.06	−1.98	−1.14	−1.58	−0.59	−0.53	−0.37	0.03	−0.15	−2.33	−0.02	−0.57	−0.32	−1.28	−0.21	−0.62	−0.12
>70 % pine	−0.46	−0.69	−0.95	−0.92	−0.76	−0.41	−0.67	−0.11	3.09	0.04	−1.81	−0.01	0.06	−0.25	−0.86	0.06	−0.49	−0.23
>50 % deciduous	−0.48	−0.43	−0.08	−1.62	−0.22	0.04	0.74	−0.41	1.04	0.08	−1.27	−0.02	0.04	−0.02	−1.09	−0.53	−0.34	1.18
20–50 % deciduous	−0.26	−0.41	−0.28	−0.56	−0.43	−0.90	−1.60	−0.10	4.96	0.13	−1.30	0.13	0.06	0.12	−0.06	−0.03	0.00	0.02
>70 % mixed conifers	−0.02	−0.20	−0.75	−0.32	−0.44	−0.02	−0.08	−0.11	−0.70	−0.03	−0.63	−0.01	−1.09	−0.23	−0.17	0.00	0.02	−0.08

*1* spring, *2* summer, *3* autumn, *4* late autumn

Clear-cuts had positive mean coefficients for many chemical variables, most notably TIN and SO_4_^2−^. Also, Cl^−^, Mg^2+^, Na^+^ and K^+^ had positive coefficients for clear-cuts, whereas Ca^2+^ did not (Table 6a). Clear-cuts also appeared as a positive factor in some of the top models for TOC, TN and TP (Table 6a). The all-season mean coefficient of 1.3 for TIN (Table 6b) corresponds to about 20 times higher TIN from a 100 % clear-cut catchment compared to a catchment without clear-cuts. For a 10 % clear-cut area, i.e. approximately the mean clear-cut area in this study (Table 3) (and Sweden), this corresponds to an approximately 35 % increase in TIN leaching, compared to growing forests. The corresponding all-season mean coefficients for the regional models (data not shown) were, on average, lower, suggesting 9 and 17 % increased TIN leaching for 10 % clear-cut catchments in the southwest and Dalälven, respectively. For TOC, TN and TP, the mean coefficients for all seasons were 0.12, 0.32 and 0.2, respectively (Table 6b), which corresponds to 2 %, 8 % and 5 % increase, respectively, in a catchment with 10 % clear-cut area. For these variables, the effect was negligible when the two regions were analyzed separately. For Na^+^, Mg^2+^ and K^+^, the mean coefficients for all seasons indicated an approximately 5-fold increase in 100 % clear-cut catchments, and there was a tangible effect of clear-cuts in both regions.

The proportion of wetland was an important predictor for many of the chemical variables. Typically, forest wetlands with a basal area >3 m^2^ ha^−1^ were more important than non-forested. Based on the best all-season models (Table 6b), the highest positive mean coefficients for forested wetlands were found for Fe (3.2), Mn (2.4) and TOC (1.3), while there were large negative coefficients for pH (−4.1) and Ca^2+^ (−1.3). The other base cations had small positive mean coefficients. TN, TP and TIN had positive mean coefficients.

For many of the chemical variables, there were negative all-season mean coefficients for the forest classes (Table 6b). This was the case for all tree classes, i.e. all of the Norway spruce, Scots pine and deciduous dominated stands, as well as the mixed forest classes. In the regional models, the negative signs often disappeared for both southwest and Dalälven (data not shown). In many cases, there were opposing signs for the dominating tree species mean coefficients between the two regions.

## Discussion

### Uncertainties

A study like this includes a long chain of various uncertainties, which, to some extent, is possible to control (water sampling, chemical analyses, forest inventories, satellite images, model selection, parameter estimation etc.), but how the uncertainty propagates through the assessment is difficult to quantify. Uncertainty related to stream water sampling is generally less than the analytical precision for the chemical analyses (Löfgren et al. 2010a), and compared with the water chemical concentration gradients in this study (Table 4), these uncertainties are of no importance. For uncertainties related to forest inventory data and remote sensing data, information can be obtained by contacting Swedish NFI (http://www.slu.se/en/webbtjanster-miljoanalys/forest-statistics/contact-us/) and Metria (http://www.metria.se/Startpage/Contact-us/), respectively.

For the probabilistic classifier method, the entropy gives uncertainty on pixel level. For a given number of classes, the entropy is maximized when all classes have the same probability. This maximum entropy increases with an increasing number of classes. By dividing the calculated entropy with the maximum entropy, comparisons can be made for different levels of aggregation, yielding an unbiased estimate (Yu and Ranneby 2007a, b). Standard statistical practice ignores model uncertainty, leading to risk for overconfident inferences and decisions. Bayesian model averaging (BMA) provides a coherent mechanism for accounting for this model uncertainty (Feldkircher and Zeugner 2009). Hence, in this study, the coefficients of determination for the best models (Table 5) are affected by the aggregated uncertainty for the entire assessment chain.

### Two distinctly different regions

The differences in concentrations between the two regions were substantial. The importance of region is also indicated by the first principal component in the PCA analysis, which explained approximately 30 % of the variation in the dataset (Fig. 2). For most water quality variables, the 25th percentile in the southwest was approximately equal to or even higher than the 75th percentile in the Dalälven region (Table 4). Similarly, there were also large variations between regions for some of the explanatory variables (Table 3). Forest growth and biomass were significantly higher in the southwest compared to Dalälven. Forest growth overlapped in the lower range 0.8–4.1 m^3^ ha^−1^ year^−1^, but with values up to 7.0 m^3^ ha^−1^ year^−1^ in the southwest, while forest biomass was typically about 30 kton ha^−1^ higher in the southwest (Table 3). In addition, the relative abundance of dominating tree species differed between the two regions, and Norway spruce was more abundant in the southwest and Scots pine more common in Dalälven (Table 3). The two distinctly different regions with different levels in stream water concentrations for most variables and landscape characteristics have resulted in models that reflect these differences.

The separation of both water chemistry and landscape data into two clusters, each representing one of the two regions, implies that any explanatory variables separating the two regions are likely to be included in the statistical models. Models with region, latitude or longitude as dependent variables show that forest characteristics (Table 2) are highly related to region and latitude (*r* ^2^ ≥ 0.8, Table 7), while longitude is less important (*r* ^2^ = 0.28). In both regions separately, the north-south location is well simulated by the models (*r* ^2^ ≈ 0.6). In Dalälven, longitude is also important (*r* ^2^ ≈ 0.5) and corresponds to temperature and precipitation gradients. The Dalälven catchment stretches from low-productive mountain forests in the west to productive managed forests in the east, resulting in a good relationship between forest parameters and longitude.

**Table 7:** Coefficient of determination (*r* ^2^ values) for the best models based on catchment properties (Table 2) from both regions and separated for Dalälven and southwest

Dependent variable	Both areas	Dalälven	Southwest
Region	0.80	Not relevant	Not relevant
Latitude	0.83	0.59	0.62
Longitude	0.28	0.54	0.36
Temperature	0.82	0.52	0.54
NH_x_ deposition	0.73	No relation	0.51
NO_x_ deposition	0.81	0.51	0.62
SO_4_ deposition	0.82	0.51	0.48

Due to these ecoregional differences, results must be interpreted with great caution. This has been dealt with by making separate models for each region and by evaluating the explanatory variables in relation to expected water chemical effects due to forest production (assimilation/mineralization of nutrients: TIN and K^+^ and formation of organic matter: TOC, TN and TP), atmospheric deposition (SO_4_^2−^, Cl^−^, Na^+^) and the share of wetlands (TOC, base cations, pH, Fe, Mn).

### Forest production

In the models presented here, the large differences in forest growth between Dalälven and the southwest have a significant role in separating the regions (Table 3). If forest growth is removed from the list of potential predictors, forest biomass becomes the most important explanatory variable. Forest growth and biomass are strongly related to each other (*r* ^2^ = 0.80). As already stated, latitude (*r* ^2^ = 0.83) or mean annual temperature (*r* ^2^ = 0.82) could also be used to separate the two different regions. Due to their geographical location, with the southwest region neighbouring the Kattegat Sea, deposition of chloride, sulphate-sulphur (*r* ^2^ = 0.82) and TIN (*r* ^2^_NO3_ = 0.81 and *r* ^2^_NH4_ = 0.73) may also separate the two regions (Table 7).

The relations with latitude and temperature are to be expected since both latitude and altitude are highly correlated to forest production and biomass. These geographical factors are used in Sweden for estimating growth both at tree and stand levels and are used as substitutes for climatic gradients (Ekö 1985; Söderberg 1986). In this study, forest biomass and production are estimated independently of latitude with the probabilistic classifier method, creating models from satellite images and ‘ground truth’ measurements by the Swedish NFI. Additionally, variations within each catchment are accounted for by the probability for each land cover, forest growth and biomass class at pixel level (Yu and Ranneby 2007a). Hence, the differences between the regions with respect to forest production and biomass are well documented. However, it remains to identify whether it is forest production per se or some other variables which are correlated with forest production that have the causal relationships with water chemistry.

When each region was analyzed separately, forest growth was not an important predictor in the southwest for some of the chemical variables, whereas it was in Dalälven. This was the case for TOC, TN, TP and K^+^, which all are variables related to biological production. For the Dalälven region, there was a relatively strong positive correlation between mean annual temperature and forest growth (*r* = 0.63) whereas this was not the case in the southwest (*r* = 0.11, Table 8). The long-term mean annual temperature varied with almost no overlap in the ranges 0.5–4.5 and 4.5–6.5 °C, respectively (Table 3). Consequently, the growing season varied between 160 and 180 days in Dalälven and between 190 and 210 days in the southwest (Table 3). Water is another important factor for forest growth and the long-term precipitation was, on average, 200 mm larger in the southwest compared to the Dalälven region (Table 3). In summary, the climatic gradients, which affect forest production, are stronger in the Dalälven region than in the southwest, possibly explaining the better models for the former area.

**Table 8:** Regression coefficients (*r* values) between selected variables and forest growth (Table 2) for both regions and separated for Dalälven and southwest

Dependent variable	Both areas	Dalälven	Southwest
Temperature	0.74	0.63	0.11
Latitude	−0.72	−0.67	0.11
Longitude	−0.31	0.56	−0.17
Altitude	−0.70	−0.84	−0.05
NH_x_ deposition	0.67	−0.05	0.08
NO_x_ deposition	0.70	0.57	−0.13
SO_x_ deposition	0.73	0.59	0.10

In Sweden, nitrogen is the most limiting nutrient for forest growth, and there is a strong relation between N availability and forest production (Binkley and Högberg 1997; Tamm 1991). Atmospheric deposition is an important N input to forest ecosystems (op. cit.). During this investigation, the atmospheric deposition of TIN varied without overlap between 8–14 and 3–5 kg N ha^−1^ year^−1^ in the southwest and Dalälven, respectively (Table 3). Due to high rates of atmospheric deposition, N fertilization is prohibited in the southwest (Swedish Forestry Act). In the Dalälven region, N fertilization was performed on <6,000 ha in 2011 (Swedish Forest Agency 2012), which corresponds to <0.3 % of the area of productive forestland. Hence, the randomly selected headwater streams in Dalälven are not likely to be affected by N fertilization to any large extent. Interestingly, there is no relation between NH_x_ deposition and forest growth in either region. Only the Dalälven area exhibited a positive correlation between forest production and NO_x_ deposition (Table 8). This indicates that the forest N demand is primarily governed by N availability coupled to N mineralization in the soils. In a short-term perspective, the annual N deposition is an extra N source for the forest ecosystem, but it does not determine the production level. In the time perspective of multirotation periods, however, atmospheric deposition must have been a major source of N to the hemiboreal landscape, strongly affecting soil productivity.

In both regions, relatively low inorganic nitrogen (TIN) concentrations during summer and autumn compared to spring and late autumn concentrations (Table 4) indicate less efficient N retention outside of the growing season. Additionally, it is evident that forests in the southwest have a more open N cycle, exporting more N to receiving streams regardless of season compared with the Dalälven region. This indicates that high forest production and enhanced losses of nitrogen to surface waters are related somehow (see below).

A positive relationship between forest growth and dissolved organic matter has previously been reported by Lauerwald et al. (2012) who found that net primary production was positively related to DOC concentrations in streams across the USA. In a Finnish study, stream water concentrations of TN, TIN and TP were positively related to stem volume in the catchment (Mattson et al. 2003). In Sweden, differences in net primary production have been suggested as a reason for decreasing soil water DOC concentrations with increasing latitude (Fröberg et al. 2006). It has also been proposed that the amount of soil organic matter in soils across Sweden is related both to temperature (Callesen et al. 2003) and N deposition (Kleja et al. 2008).

For TIN, K^+^, OrgN and TOC, forest growth per se is likely not a factor that has a significant direct effect on stream water chemistry. Instead, the elevated concentrations of many elements at sites with high growth can probably be attributed to factors simultaneously affecting forest production and stream water chemistry. Climate (temperature and precipitation), extensive soil element pools and N deposition are the most likely candidates. Results presented here support a hypothesis that the potential losses of organic species and inorganic nutrients is higher in nutrient-rich stands with a climate favouring leakage compared with stands of lower fertility and a climate less conducive to element losses. However, the mechanisms may differ between water chemistry variables depending on whether they are produced (DOC, TON) or assimilated (TIN, K^+^) by vegetation.

In the southwest where winter temperatures are often above 0 °C and precipitation primarily in the form of rain, the prerequisites are created for high groundwater levels and leakage of organic matter to streams throughout the year. At base flow, during summer and autumn, peatlands and riparian soils may be important sources of organic matter (Löfgren and Cory 2010; Winterdahl et al. 2011), yielding high concentrations of TOC, TN and TP (Table 3). In the Dalälven region, winter precipitation (December to March) generally accumulates as snow with few or no melt events, creating continuously descending groundwater levels and decreasing TOC concentrations in run-off. This is further accentuated in spring, when large volumes of snowmelt water may dilute mobilized organic matter (Table 3). As in the southwest, organic matter is derived primarily from wetlands during base flow.

A high nutrient demand during the growing season makes TIN (and K^+^) less available for leakage, while winter, early spring and late autumn are potential periods for tangible losses (Tables 3 and 4). Both net mineralization and atmospheric deposition may contribute to excess TIN (and K^+^) concentrations in run-off during the dormant season. The atmospheric N deposition is much higher in the southwest compared with Dalälven, and from the forest production figures, it could be assumed that mineralization is too. The prerequisites for higher TIN concentrations in the southwest (Tables 3 and 4) are therefore fulfilled.

The models reflect two distinctly different regions, and the influence of dominant tree species should therefore be interpreted with great caution. For many chemical variables, there was a negative influence of all dominant tree species in the model for both regions combined (Table 6b). This negative influence usually disappeared in the separate regional models (Table 6a). There is a complex multifactor interaction between forest production and the amount of Norway spruce and Scots pine. Forest growth increases, e.g. with Norway spruce in the southwest, but not in Dalälven. If region is used as response variable, the relations are positive for forest growth and negative for these two tree species. Based on an ANOVA, forest growth and Scots pine are significantly related (*p* < 0.05) as well as the three-way interaction between forest growth, Scots pine and Norway spruce. This suggests that the negative influence of dominant tree species on the chemical variables in the models should be interpreted as statistical artefacts rather than real effects.

### Forest management

Clear-cuts are well known to result in increased N concentrations in soil water (e.g. Akselsson et al. 2004; Futter et al. 2010) and surface waters (e.g. Löfgren et al. 2009b; Rosén et al. 1996). Where in the catchment, clear-cutting occurs and the existence of buffer zones along a stream may also be important factors for N and P export (Löfgren et al. 2009b). On a large scale, however, the effects of clear-cuts on the nutrient loads to the sea surrounding Sweden are low (Brandt et al. 2009; Futter et al. 2010).

The data presented here suggest a strong increase of TIN after complete clear-cutting, which generally includes also soil tillage and planting (see Introduction), of a headwater catchment. Mean coefficients for the whole dataset (Table 6b) suggest as much as a 20-fold increase at 100 % clear-cut compared with a non-harvested area (mean coefficient for clear-felling = 1.3, log_10_TIN = 1.3*clear-cut), although this figure is not well constrained and is considerably lower in the regional models. On a larger scale, some fraction of the total N and P loads from forests to the sea may be attributed to clear-cutting. In 2006, 4.3 % of the forestland in the southwest region which drains to the Kattegat was clear-felled (Brandt et al. 2009). Based on the all-season best model, this corresponds to a TIN concentration increase of 10 %. For the Kattegat, Brandt et al. (op. cit.) estimated a 10 % increase in gross nitrogen load from forestland due to increased TIN leakage from clear-felled areas. Changed run-off after clear-cutting was not taken into account in these load estimates (op. cit.), implying a 10 % TIN concentration increase.

In other studies where large fractions of small catchments have been clear-cut, there have been significant effects on C, N and P in streams (Löfgren et al. 2009b). Vuorenmaa et al. (2002) reported 40–70 % higher P and a more than 3-fold increase in N after clear-cutting 80 % of a catchment in Finland. Hence, on a headwater catchment scale, the impact on stream water concentrations may be significant if a large proportion of the catchment is harvested within a short period of time. In the data presented here, up to about half of the area of a single catchment was classified as clear-cut. The results for TOC in relation to clear-cuts are similar in magnitude to those observed by Laudon et al. (2009), who reported an up to 50 % increase in stream water DOC concentrations after harvesting large parts of boreal catchments in Northern Sweden. Mean coefficients (Table 6b) in this study suggest an increase of about 30 % in TOC concentration after complete harvest. Thus, the contribution from clear-cuts to total transport of TOC on a large scale (i.e. clear-cut area ~10 %) was estimated to be only a few percent, but the effect on a smaller scale may be significant if large portions of the catchment are subjected to harvest.

Removal of base cations with trees is another effect of forest harvest, potentially contributing to acidification of soils and water (Zetterberg et al. 2013). Increased leaching of base cations reinforces the negative effect of clear-cutting (Titus et al. 1998). In this study, positive relationships between clear-cuts and Na^+^, K^+^ and Mg^2+^, but not Ca^2+^, were found. Mean coefficients (Table 6b) suggest a 10–20 % increase in base cation concentrations from forest catchments on a large scale (i.e. with about 10 % of the area clear-cut) as a result of clear-cutting in Sweden. In addition, SO_4_^2−^ had positive mean coefficients for clear-cuts. The SO_4_^2−^ leakage and higher NO_3_^−^ loss cause increased base cation leaching in order to maintain ion balance in solution (Zetterberg et al. 2013). Kreutzweiser et al. (2008) concluded in a review that forest harvest might result in increased leaching of base cations. Rosén et al. (1996) found distinct increases in K^+^, inorganic and organic N in experimentally clear-cut catchments in Central Sweden, accompanied by less distinct increases for Na^+^, Ca^2+^, Mg^2+^, Cl^−^ and SO_4_^2−^. Increased leakage of K^+^ but no effects on Ca^2+^ was also found after clear-cutting in northern Sweden (Löfgren et al. 2009b).

Following harvest and regeneration in the studied regions, the most extensive forest operation is thinning on one or more occasions. N fertilization, ash treatment, etc. are rare or prohibited. The relations between such activities and water quality have not been evaluated per se, but the effects of thinning operations are included in the production and biomass estimates. As already stated, production per se does not seem to have a causal relation with water quality, indicating few or negligible effects of thinning.

### Atmospheric deposition

Based on all data, the stream water variables that gave best model fits (Table 6b) were Na^+^, Cl^−^ and SO_4_^2−^ (0.6 < *r* ^2^ ≤ 0.8, Table 5), which are all known to be closely related to atmospheric deposition (Löfgren et al. 2010b). As already shown, forest growth, temperature, precipitation and atmospheric depositions are closely correlated across Sweden, and good fits for these variables are therefore expected. In the two separate regions, the model fits were lower (*r* ^2^ ≈ 0.5, Table 5), but still the best among the studied water chemical variables. The good model fits for these constituents, poorly coupled to forest growth, strongly support a conclusion that it is not forest production per se that causes increased organic matter and nutrient concentrations in stream water.

### Wetlands

The proportion of forested wetland (basal area ≥3 m^2^ ha^−1^) was included as a positive factor in models for TOC, TN, TP, Fe and Mn and as a negative factor for Ca^2+^, pH and ANC (Table 6). The importance of wetlands is also indicated by the second principal component in the PCA analysis, explaining approximately 15 % of the variation in the dataset (Fig. 2). There were also some tendencies for a positive relationship for non-forested wetlands (basal area <3 m^2^ ha^−1^), for the aforementioned variables and for Na^+^, K^+^, Mg^2+^ and TIN (Table 6b). Higher concentrations of dissolved C, N and P in streams from catchments with large contributions from wetlands have repeatedly been shown in other studies (e.g. Dillon and Molot 1997; Laudon et al. 2004; Johnston et al. 2008; Lauerwald et al. 2012). The positive effect of wetlands on these variables was observed in the models for both regions and in the separate regional modelling. There was also a logical sequence for the influence of wetlands in the models (Table 6). Wetlands had the largest influence on TOC (100 % organic), followed by TN, which is dominated by organic species and then by TP, which includes both organic P, but also a significant fraction of inorganic P. For TIN, the connection to wetlands was weak.

Fe and Mn are two redox sensitive elements, and the positive relationship with wetlands is in agreement with catchments in Ontario, Canada (Dillon and Molot 1997), New York, USA (Maranger et al. 2006), and Finland (Kortelainen et al. 2006). The negative relationship between wetlands and Ca^2+^ is explained by the strong connection to mineral weathering for this element and the restricted hydraulic connectivity with minerals in peat-dominated areas. The negative relation between wetlands and ANC, which also affect pH, is a result of lower base cation concentrations. Besides ANC, pH is also affected by organic acidity derived from peat.

## Conclusions

This assessment indicates that element losses from forest-dominated mosaic landscapes are related to factors associated with forest production but that does not necessarily mean that it is the forest production per se that causes the excess losses. Instead, factors simultaneously affecting forest production and stream water chemistry, with climate (temperature and precipitation), extensive soil pools and N deposition, are the most likely candidates for the water chemical gradients.

However, some relationships between catchment properties, forestry practices and water chemistry are likely to be direct effects. This is shown by clear differences in explanatory variables between the chemical models and the model of region. Neither wetlands nor clear-cuts were important factors for modelling of region (Table 6) but are included in many of the models for stream water chemistry, thus suggesting an actual effect of these factors on the water chemistry (e.g. TOC, TN, TIN, TP, pH and ANC).

Hence, the forestry sector could use this type of models in order to identify water bodies where special precautions are necessary and water authorities could improve the estimates of element leakage from mosaic landscapes and to make more accurate source apportionments, thereby quantifying the possibilities to reduce e.g. nutrient loads to the sea. In the latter case, however, water discharge and between-years variation are necessary to take into account. Currently, the nutrient reduction targets within the Baltic Sea Action Plan (HELCOM) have been criticized for not properly estimating the different background leakage from different land cover, causing unachievable reduction targets in e.g. hemiboreal areas (Swedish Farmers 2013).

In the context of forest management, these results indicate that focus should be on improving harvesting techniques at clear-felling and associated regeneration practices such as soil tillage in order to reduce negative impacts on the headwater stream chemistry. At least at a local scale, this may improve water quality. The effects of thinning operations, performed later in the forest succession, seem to be low or negligible.
